# Additional value of a dynamic contrast-enhanced study for detection of a small neuroendocrine tumor of the rectum on magnetic resonance imaging

**DOI:** 10.1590/0100-3984.2017.0150

**Published:** 2019

**Authors:** Leandro Lucas Lima, Daniella Braz Parente, Ricardo Vezzani Batista, Antonio Eiras de Araújo

**Affiliations:** 1 Universidade Federal do Rio de Janeiro (UFRJ), Rio de Janeiro, RJ, Brazil.; 2 Instituto D'Or de Pesquisa e Ensino, Rio de Janeiro, RJ, Brazil.

*Dear Editor,*

**Figure 1:**
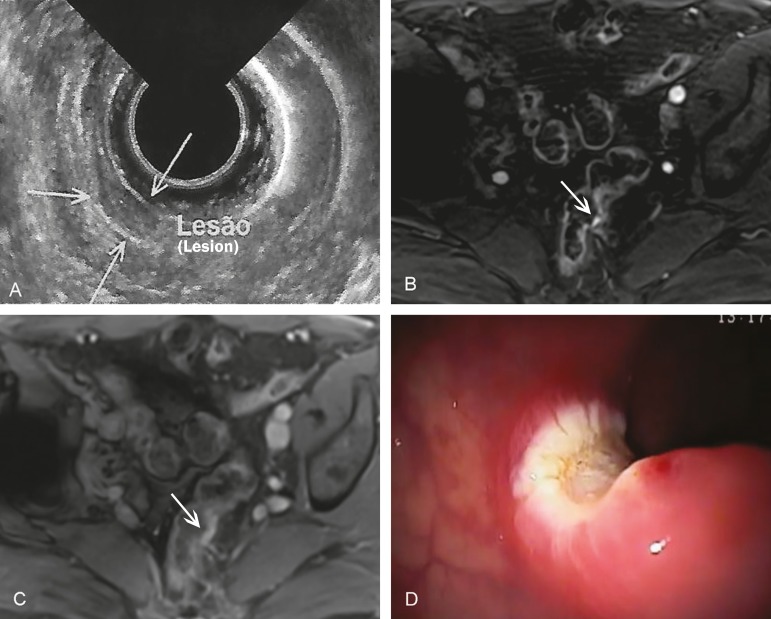
**A:** Endoscopic ultrasound showing a localized lesion restricted to the submucosa (arrows). Contrast-enhanced 3.0 T MRI with a dynamic study (**B**), showing a small, hypervascular parietal lesion in the early phase (arrow) and in the late stage (**C**) indicating that the lesion was no longer isolated (arrow). **D:** Eschar resulting from the resection.

Screening colonoscopy revealed a subepithelial lesion in the rectum of a 70-year-old asymptomatic man, a finding that was subsequently confirmed by endoscopic ultrasound (Figure 1A). The patient then underwent magnetic resonance imaging (MRI), performed in accordance with the routine protocol, on which the lesion was not detected. An MRI scan was complemented with a dynamic study, which revealed a 5 mm lesion that showed contrast enhancement in the early phase and no enhancement in the later phases (Figure 1B,C). Subsequently, endoscopic ultrasound was performed for diagnostic and therapeutic purposes, including resection of the lesion (Figure 1D), the histopathological diagnosis of which was a differentiated neuroendocrine tumor. 

Neuroendocrine tumors can occur in various organs, and they account for 1.5% of all gastrointestinal or pancreatic neoplasms^(^^1^^)^. In the gastrointestinal tract, the rectum is the second most commonly affected region, accounting for 21-27% of cases^(^^2^^)^. Although most neuroendocrine tumors are idiopathic, up to 25% are associated with genetic syndromes such as multiple endocrine neoplasia type 1, neurofibromatosis type 1, von Hippel-Lindau disease, and tuberous sclerosis^(^^3^^-^^5^^)^. They can produce hormones and metabolically active amines, resulting in symptoms^(^^5^^)^. Nonfunctioning neuroendocrine tumors, which are more common, frequently appear as locally advanced disease or as metastasis, especially to the liver^(^^5^^)^. The World Health Organization 2010 classification system utilizes histopathological aspects to classify neuroendocrine tumors into three categories: grade 1; grade 2; and grade 3. Grade 1 neuroendocrine tumors are benign or of uncertain malignant potential, whereas those of low malignant potential are categorized as grade 2 and those that exhibit aggressive behavior are categorized as grade 3^(^^6^^)^.

The rate of malignancy for neuroendocrine tumors depends on their histological classification and site of origin. When they are located in the rectum, the prognosis is good. Most patients with rectal neuroendocrine tumors are asymptomatic and have small lesions (< 1 cm) that are discovered incidentally and are localized at diagnosis in 82% of cases, metastasis occurring in only 2% of those < 2 cm, and the five-year survival rate is 88%^(^^2^^)^. Endoscopic ultrasound is the ideal method to evaluate lesions invading the rectal wall, as well as to evaluate the regional lymph nodes^(^^7^^)^. MRI has come to be ever more widely used for evaluating the extent of the tumor and nodal involvement^(^^2^^)^. The ideal treatment is local resection for noninvasive lesions < 2 cm and radical surgery with resection of the draining lymph nodes for invasive lesions or for those > 2.5 cm^(^^8^^)^. For metastatic tumors, systemic or even surgical therapies are considered^(^^2^^)^.

Differentiated rectal neuroendocrine tumors originate from the muscle mucosa or submucosa and are therefore located superficially or have intraluminal growth patterns. They present hypointense signals on T1-weighted sequences, and hyperintense signals on T2-weighted sequences, with homogenous contrast enhancement. However, undifferentiated neuroendocrine tumors present findings similar to those of rectal adenocarcinoma^(^^9^^)^.

Including a dynamic study increases the sensitivity of MRI for the detection of small neuroendocrine tumors. This protocol could represent a complementary method for investigating occult neuroendocrine tumors.
